# Kyphoplasty as a Treatment Option for Traumatic Burst Fractures: A Case Series Evaluating Patient Outcomes and Functional Benefits

**DOI:** 10.3390/brainsci15060659

**Published:** 2025-06-19

**Authors:** Anoop S. Chinthala, Barnabas Obeng-Gyasi, Trenton A. Line, Matthew K. Tobin, Gordon Mao, Bradley N. Bohnstedt

**Affiliations:** Department of Neurological Surgery, Indiana University School of Medicine, 355 W 15th St., Suite 5100, Indianapolis, IN 46202, USA; aschinth@iu.edu (A.S.C.); bobenggy@iu.edu (B.O.-G.); treline@iu.edu (T.A.L.); mktobin@iu.edu (M.K.T.); gomao@iu.edu (G.M.)

**Keywords:** kyphoplasty, thoracolumbar burst fracture, pain

## Abstract

**Background/Objectives:** Kyphoplasty and vertebroplasty are minimally invasive approaches for spinal fractures aiming to reduce pain, increase mobilization, and prevent further vertebral height loss. Their efficacy in treating burst fractures has been questioned due to fragment mobility and concerns for cement leakage. We aim to report outcomes in patients who underwent kyphoplasty for spinal burst fractures. **Methods**: We conducted a retrospective review of patients with burst fractures treated from 2018 to 2023. Those who underwent kyphoplasty or vertebroplasty and had follow-up imaging were included. Clinical characteristics and follow-up outcomes were obtained through chart review. The primary outcome was the need for surgical intervention after kyphoplasty. **Results**: We identified ten patients (mean age 67.9 years, range 36–93 years) with burst fractures who underwent kyphoplasty/vertebroplasty. Six received kyphoplasty/vertebroplasty within 1 week of injury and four between 1 and 4 months post-injury. Nine patients had a TLICS score of 2, and one had a TLICS score of 5. Kyphoplasty/vertebroplasty was performed for pain management in seven patients and significant/worsening vertebral height loss in three patients. At follow-up, 70% of patients reported an improvement in pain and 75% of patients reported improved mobility. One patient experienced progression of an L2 burst fracture but improved with conservative management. No patient required additional surgical fixation. **Conclusions**: In this series of ten patients with spinal burst fractures, standalone kyphoplasty was a safe and effective treatment. Our findings suggest kyphoplasty may be a viable treatment option for select spinal traumatic burst fractures, offering potential pain relief and mobility improvement in the short term.

## 1. Introduction

Burst fractures are a type of a spinal compression fracture involving the anterior and middle columns of the spine. These fractures most commonly occur at the thoracolumbar level and raise concern for possible neurological injury due to bony retropulsion or anatomic instability due to kyphotic deformity. There is also concern for the progression of kyphotic deformities. The management of burst fractures varies depending on their classification, clinical presentation, and radiological findings, ranging from conservative treatment to surgical intervention. Various surgical strategies exist to approach these lesions, although no clear consensus exists on the optimal approach [1].

Minimally invasive spine surgery techniques, such as percutaneous pedicle screw fixation and kyphoplasty, have emerged as alternatives to traditional open surgery, offering reduced morbidity and mortality. Kyphoplasty has shown promise in treating thoracolumbar burst fractures in both neurologically intact and impaired patients, demonstrating significant pain reduction and kyphotic correction [2,3,4,5,6]. This minimally invasive procedure involves creating a cavity within the fractured vertebral body using an expandable balloon, which is then filled with bone cement. Historically, the use of bone cement in fractures involving the posterior wall was contraindicated due to the risk of cement leakage; however, recent advances have shown that cement leakage rates are low in kyphoplasty with instrumentation for traumatic burst fractures [7,8]. Furthermore, most of the data reported is in patients that received cement augmentation in addition to standard pedicle screw fixation. Therefore, very little is known about the utility of standalone kyphoplasty for the management of acute thoracolumbar burst fractures [8,9,10,11,12,13].

Kyphoplasty without pedicle instrumentation has gained attention as a treatment option for spinal burst fractures, particularly in osteoporotic patients [14]. While many studies have reported satisfactory clinical outcomes, including pain reduction, rapid functional recovery, and restoration of vertebral height, the majority of these reports involve kyphoplasty combined with pedicle instrumentation [8]. There is limited data on the use of standalone kyphoplasty for the treatment of traumatic burst fractures. Most existing studies focus on osteoporotic fractures and exclude traumatic burst fractures or only focus on incomplete or type A3 burst fractures [15]. Additionally, the few studies that have examined kyphoplasty without instrumentation in traumatic burst fractures often involve augmented techniques, such as armed kyphoplasty with interverbal implants [8].

To our knowledge, no prior case series has analyzed the use of standalone kyphoplasty for the treatment of traumatic incomplete and complete burst fractures. Our objective is to examine the safety, pain reduction, and mobilization of standalone kyphoplasty in a series of patients with traumatic burst fractures.

## 2. Materials and Methods

**Figure 1:**
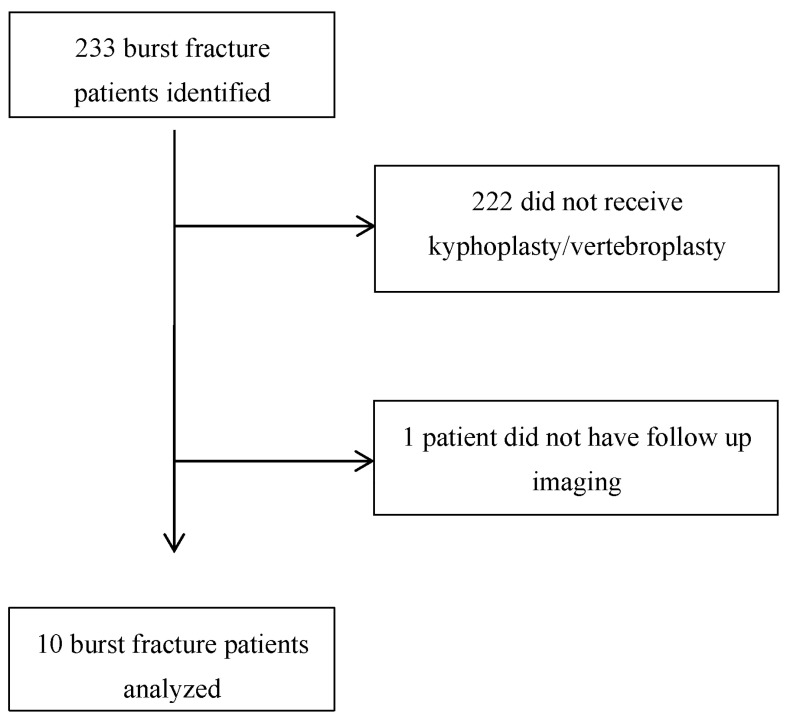
Flow chart of excluded patients.

We retrospectively collected data using Dig Our Radiology Information System (DORIS; an internal institutional database collating all radiology reports across all sites from our institution) on patients who presented with burst fractures between 1 January 2018 and 31 December 2023. Our inclusion criteria included patients ≥ 18 years old with radiographically confirmed burst fractures that received a kyphoplasty or vertebroplasty. We excluded patients lacking follow-up images. A total of 233 patients were identified to have burst fractures. Of these patients, only 11 patients received kyphoplasty and 10 patients had follow-up imaging (Figure 1). The following variables were collected for each patient: demographic information, medical comorbidities (presence of cardiac conditions, chronic kidney disease, osteoporosis, and other chronic illnesses), injury characteristics, American Spinal Injury Association (ASIA) Impairment scale, Thoracolumbar Injury Classification and Severity Scale, and indication for procedure. Interval (days) was calculated based off when the initial injury occurred and the date of the kyphoplasty. Follow-up information was collected using data from neurosurgery clinic notes at the 1 month follow-up appointment. These variables the included presence of neurological deficit, cement leakage or extravasation, and whether the patient described pain or mobility improvement. Radiographic X rays were reviewed by independent board-certified radiologists as well as by the treating practitioners to identify stability or any complications post procedure. A failed kyphoplasty was defined as the need for subsequent spinal surgery or invasive procedure to address the original injury treated by the kyphoplasty.

Admission and preoperative Computed Tomography (CT), Magnetic Resonance Imaging (MRI), and X-ray imaging were reviewed. Variables collected included kyphotic angle, retropulsion, anterior–posterior canal diameter, vertebral body height loss, interpedicular distance, and residual canal area. All imaging studies were independently reviewed by a fellowship-trained spine neurosurgeon. Radiographic measurements were conducted using CT scans to assess key parameters related to the fractures. Kyphotic angle was measured on mid-sagittal CT slices at the fracture level by focusing on the AP limits of the superior and inferior endplates calculating the angle between lines drawn along the superior and inferior endplates of the fractured vertebra. Retropulsion of bone fragments was quantified using both mid-sagittal and axial CT images. On sagittal slices, the maximum posterior displacement of fragments was measured from the posterior vertebral body line, while on axial cuts, the horizontal displacement of the fragment into the spinal canal was assessed. The AP canal diameter was measured using the smallest canal diameter between the posterior vertebral body line and the spinolaminar line. Vertebral body height loss was calculated by comparing the anterior height of the fractured vertebra, expressed as a percent loss. Interpedicular distance was assessed on the axial CT images as the horizontal distance between the medial aspects of the pedicles the level of maximum retropulsion, while residual canal area was measured at the level of maximal stenosis.

## 3. Results

### 3.1. Illustrative Case

A 76-year-old female with a past medical history significant for diabetes mellitus presented to an outside hospital after a mechanical fall from a step stool at home. Imaging at the outside hospital demonstrated an L1 burst fracture, and she was subsequently transferred to our main facility. On presentation, her numeric pain scale rating was 10/10 in the lower back without radiation. She denied numbness, tingling, or weakness. Physical examination revealed intact strength and sensation, no clonus, negative Hoffmann’s sign, and +2 deep tendon reflexes.

**Figure 2:**
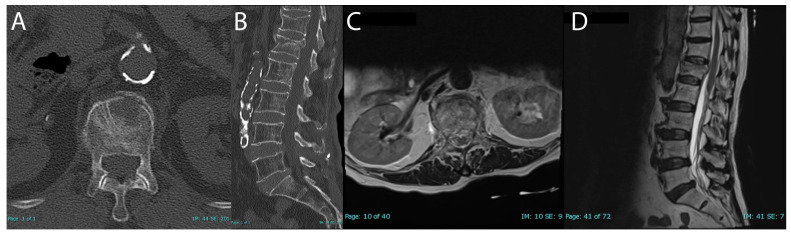
Preoperative CT and MRI assessment of burst fracture (**A**–**D**); admission CT axial (**A**); CT sagittal (**B**); admission MRI axial T2 (**C**); MRI sagittal T2 (**D**).

CT and MRI confirmed an acute two-column L1 vertebral compression fracture with approximately 26% height loss and 4.3 mm of retropulsion (Figure 2). Additionally, there was evidence of a mild chronic T12 compression fracture. MRI did not reveal ligamentous injury.

For pain management, she received PRN hydromorphone 1 mg every 2 h and PRN acetaminophen 650 mg orally every 4 h. It was determined to continue with pain management and conservative management with TSLO brace. On the second day of admission, the patient continued to report severe back pain, rated as 8/10, despite medical management. Given the patient’s intact status and lack of ligamentous injury or neural element compression on the MRI, we did not feel a large operation to stabilize the spine or decompress the spinal canal was necessary. As such, after discussion with the patient, we offered a standalone kyphoplasty to address her pain.

Kyphoplasty was performed without complications. Within 24 h postoperatively, her pain improved to 6/10. Postoperative pain management included PRN hydromorphone 1 mg IV push 2 h and PRN acetaminophen 650 mg orally every 4 h. She was discharged on postoperative day 2 to home with 24 h assistance and with a numeric pain scale rating of 0/10.

**Figure 3:**
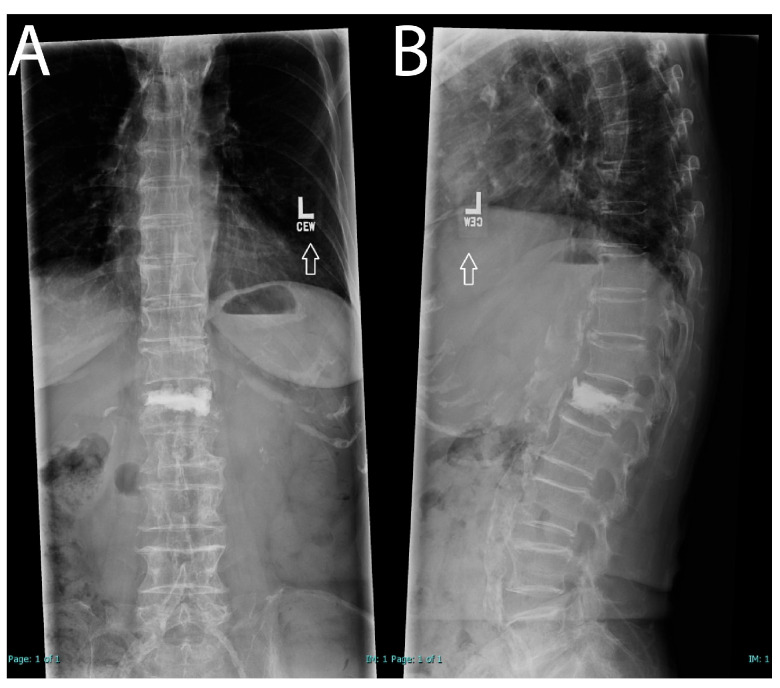
One-month postoperative X-ray (**A**,**B**); X-ray AP (**A**); X-ray lateral (**B**). Arrowheads are markers from the original imaging acquisition and are not present for demonstration purposes.

At one-month follow-up, she reported significant improvement in her pain, stating it was much better, with only mild soreness when lying in bed. She noted that she continued to wear her brace but only when leaving the house. Her husband also confirmed a marked improvement in her condition. Based on her progress, she was instructed to discontinue the use of the brace and gradually resume her normal activities. Follow-up X-ray showed stable cement in the vertebral body and no acute burst fracture (Figure 3). No further follow-up was deemed necessary. After chart review, no additional spinal operations were found after follow-up.

### 3.2. Series Results

Ten patients with thoracolumbar burst fractures were treated with kyphoplasty or vertebroplasty alone. The cases are summarized in Table 1, Table 2 and Table 3. The average age of patients was 68.6 years (range 29 to 93), and 60% were female. The mechanism of injury included fall from standing or elevated height in six patients and motor vehicle collision (MVC) in four patients. No patients had neurological deficits and all were classified as ASIA E. All patients had a TLICS of 2, with the exception of one patient that had a TLICS of 5 due to the presence of posterior ligamentous complex (PLC) injury. Eight patients had an incomplete burst fracture and two patients had complete burst fractures. All burst fractures were two column injuries. One patient presented with burst fractures on multiple levels; all other patients had burst fractures on one level. The median percentage height loss was 10.5% (range 0–29). The median kyphotic angle was 7.5 degrees (range 0–18). The median retropulsion was 5 mm (range 3–7). The median AP canal diameter was 10 mm (range 10–15). The median interpeduncular distance was 24 mm (range 21–27). The median residual canal area was 196 mm^2^ (range 163–318).

Seven patients received a kyphoplasty due to significantly worsening pain; three patients received it due to progressive height loss. Six patients had their kyphoplasty performed within one week of injury, one patient had the procedure one month from injury, and the remaining three patients received their procedure more than one month from injury. There were no intraoperative complications. Only one patient was discharged to an acute rehab facility. Two patients were discharged to a subacute rehab facility and seven were discharged home.

At one-month follow-up, there were no new reported neurological deficits, nor cement leakage or extravasation. Seven patients reported improvements in pain. Eight patients had impaired mobility due to the thoracolumbar burst fracture. Of these eight patients, six patients stated their mobility had improved after kyphoplasty.

**Figure 4:**
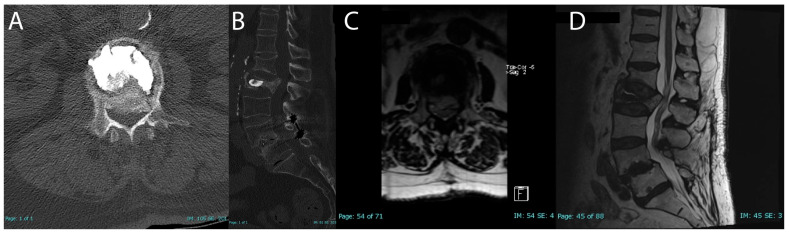
Follow-up complication for case 2 (**A**–**D**); one-month postoperative CT axial (**A**); CT sagittal (**B**); one-month postoperative MRI axial T2 (**C**); MRI sagittal T2 (**D**).

On follow-up upright standing X-rays, only one patient had a potentially unstable finding of possible settling of fracture around cement. One-month follow-up MRI showed significant settling of the L2 burst fracture with retropulsion into the canal with significant central lateral recess stenosis (Figure 4). The patient had no significant mobility issues. The pain was tolerable but was not improving. The patient was offered spinal surgery as well as conservative management for the pain. The patient chose to manage with injections and physical therapy and at three-month follow-up, the patient’s pain and mobility had improved.

Review of medical records showed that no patient in this series underwent further surgical intervention or invasive procedure for their lower back or fracture site.

## 4. Discussion

In this retrospective case series, we evaluated the clinical and radiological outcomes of standalone kyphoplasty and vertebroplasty for traumatic thoracolumbar burst fractures in ten patients. This study is one of the first to examine standalone kyphoplasty and vertebroplasty for traumatic burst fractures across various subtypes (incomplete and complete). Our results demonstrate that kyphoplasty without instrumentation can be a safe and effective treatment option for patients with traumatic thoracolumbar burst fractures presenting without neurological deficit. However, given our follow-up period was limited to one month, our findings primarily reflect early postoperative outcomes, and long-term stability cannot be conclusively determined from this data. To supplement this limitation, patient charts were retrospectively reviewed up to the present day to assess whether any individuals required reoperation for their original injury.

Adjacent pedicle screw fixation is widely regarded as the gold standard for stabilizing burst fractures; however, the specific technique and extent of fixation is debated [8,16,17]. Due to the disruption of the posterior wall in burst fractures, patients are susceptible to retropulsion of fragments which, if not treated correctly, can lead to worsening neurological function, significant vertebral height loss, and increased kyphotic angle. Indirect decompression via ligamentotaxis can be utilized intraoperatively to restore vertebral body height and potentially reduce bone fragments from the spinal canal [18]. This is achieved by applying distraction forces to cause the posterior longitudinal ligament to tighten, thus reducing the fragments back into the vertebral cavity. Consequently, many studies suggest that the addition of pedicle screw fixation, even in cases where kyphoplasty achieves direct stabilization, provides enhanced protection and facilitates improved healing. For example, He D et al. conducted a randomized controlled trial in 2013 that supported the superiority of combined kyphoplasty and pedicle screw fixation [7]. However, kyphoplasty techniques have significantly advanced in the last decade, warranting a re-evaluation of standalone kyphoplasty in this context [19].

In our institution, we use a combination of both the radiographic findings and the patient’s neurological status to determine whether they would be an appropriate candidate for a standalone kyphoplasty. In patients who are both intact neurologically and lack imaging findings concerning for posterior ligamentous injury (i.e., a stable fracture) or neural element compression, we would offer a standalone kyphoplasty procedure. Indications for kyphoplasty would include significant mechanical pain that is not responsive to conservative measures including bracing and multimodal pain therapy, as well as an increase in focal segmental kyphosis or vertebral body height loss on weightbearing X-rays. Furthermore, in patients who are not neurologically intact, we do not offer standalone kyphoplasty as we favor open surgical fixation and decompression to alleviate ongoing compression.

Given the success of standalone kyphoplasty in the treatment of osteoporotic compression fractures, there could be a potential role of similar treatment in specific subsets of traumatic burst fracture patients. Patients that present with no neurological deficits, classified as ASIA E, and two column injuries with no facet disruption could be considered candidates for standalone kyphoplasty. This approach directly addresses the patient’s symptom and mobility burden, potentially facilitating a faster recovery. A group in Germany studied standalone kyphoplasty in traumatic A3 (incomplete) burst fractures and provided promising results that standalone kyphoplasty is safe and can lead to immediate pain reduction with reduced operative risks [15]. Our theory is that by immediately stabilizing the fracture with kyphoplasty, patients are more willing and able to engage in physical therapy (PT) and other aspects of their care. Pain relief and reduced movement burden allow patients to participate more effectively in PT, which could promote earlier mobilization and expedite discharge to rehabilitation programs. Radiographic evaluation at one month could determine whether additional screw fixation is needed based on symptoms and imaging findings.

Our findings suggest that standalone kyphoplasty could provide significant clinical benefits, as 70% of patients reported improved pain at one-month follow-up, and 75% of patients noted improved mobility. This aligns with prior studies reporting pain reduction and functional recovery in patients treated with kyphoplasty with or without additional instrumentation [8,15,20,21]. All patients were deemed to have a successful standalone kyphoplasty as none required further operative treatment.

Our study included patients that received kyphoplasty/vertebroplasty at various timelines since injury. There were four patients that received kyphoplasty/vertebroplasty greater than one month from the initial injury. Initially treated conservatively, three of the four patients experienced worsening pain and were offered the minimally invasive procedure, after which their pain improved. The patient that did not experience pain improvement had a primary indication for treatment of height loss with kyphoplasty. These results indicate kyphoplasty may benefit patients in the subacute phase after failing conservative management.

Importantly, only one complication was recorded. Case 2 presented to their follow-up appointment at one month postoperative with severe left hip and thigh pain, which was later attributed to a progressive settling of the fracture and retropulsion of bony fragments into the spinal canal. CT and MRI findings confirmed that there was moderate to severe canal stenosis at L1–L2 with worsening retropulsion (Figure 3). Despite these findings, the patient’s mobility remained stable but pain was causing significant distress. After being offered both surgical and conservative options, the patient chose conservative management with physical therapy and epidural steroid injections, which led to a gradual improvement in pain and functionality over the next two months. After two sessions, the patient no longer required assistance with ambulation and was pleased with the improvement in pain, withdrawing from any further evaluation. Chart review revealed the patient did not have any further injections or spinal surgery after her last follow-up appointment.

Future studies with larger cohorts and longer follow-up periods are needed to further validate these findings and determine the long-term efficacy of standalone kyphoplasty/vertebroplasty. Comparative studies evaluating standalone kyphoplasty versus other treatment modalities, such as kyphoplasty with instrumentation and open surgical fixation, could provide valuable insights into the optimal management of thoracolumbar burst fractures.

### Limitations

Due to the retrospective nature of the study, only information in the medical records was available to us and is subject to potential biases such as patient selection for treatment with kyphoplasty/vertebroplasty. Patients were not contacted in this study and thus could have had surgical procedure or invasive surgery for their fracture at an outside hospital. Our results are limited as we were unable to obtain objective movement measures. The small sample size and observational nature of the study limits the generalizability of our results and prevents definitive claims about efficacy and safety. The follow-up period was relatively short, and long-term outcomes of radiological parameters, such as progressive kyphosis, were not assessed.

## 5. Conclusions

In this case series of 10 patients with traumatic thoracolumbar burst fractures, standalone kyphoplasty provided safe and effective treatment, offering pain relief and functional recovery with minimal complications in short-term follow-up. Our findings suggest that standalone kyphoplasty could be a viable alternative to more invasive and timely surgical approaches, particularly in patients without neurological deficits.

## Figures and Tables

**Table 1:** Demographic and clinical characteristics.

	Age	Sex	Comorbidities	Etiology	ASIA	TLICS	Procedure	Procedure Indication ^#^
Case 1	59	F	Obese, CAD, CKD Stage 5, HTN, DM, OSA, HLD	Fall from standing	E	2	Verteobroplasty	Pain
Case 2	84	F	Obese, Osteoarthritis	Fall from elevated height	E	2	Vertebroplasty	Pain
Case 3	93	F	HTN, Hypothyroidism, DM, HLD, Chronic Venous Insufficiency	Fall from standing	E	2	Kyphoplasty	Pain
Case 4	36	F	Obese	MVC	E	2	Kyphoplasty	Pain
Case 5	62	M	Heart Disease, COPD, Osteoarthritis	MVC	E	2	Kyphoplasty	Worsening height
Case 6	68	M	HTN, HLD, CVA	MVC	E	2	Kyphoplasty	Significant height loss T12
Case 7	29	M	OSA	MVC	E	2	Kyphoplasty	Pain
Case 8	76	F	DM	Fall from elevated height	E	2	Kyphoplasty	Pain
Case 9	84	M	PVD, A-Fib, Osteoarthritis, COPD, HLD	Fall	E	5	Kyphoplasty	Pain
Case 10	88	F	DM, HLD	Fall	E	2	Kyphoplasty	Height loss > 50%

Abbreviations: A-Fib—atrial fibrillation; CAD—coronary artery disease; CKD—chronic kidney disease; COPD—chronic obstructive pulmonary disease; CVA—cerebrovascular accident; DM—diabetes mellitus; HLD—hyperlipidemia, HTN—hypertension; OSA—obstructive sleep apnea; PVD—peripheral vascular disease. ^#^ Indications for procedures are listed as in the table. For all patients, larger surgeries (e.g., arthrodesis, open reduction internal fixation, instrumentation) or mixed approaches (cement augmentation with open surgery) were not indicated in any of the patients due to lack of posterior ligamentous injury and lack of neural element compression confirmed by MRI and intact neurological status.

**Table 2:** Preoperative radiological and fracture characteristics.

	Days from Injury to Kyphoplasty	Fracture Level	% Height Loss	Kyphotic Angle	Retropulsion (mm)	AP Canal Diameter (mm)	Interpeduncular Distance (mm)	Residual Canal Area (mm^2^)	Burst Fracture Type
Case 1	6	L1	29	15	7	10	21	165	Incomplete
Case 2	6	L2	12	0	5	10	25	196	Complete
Case 3	4	T11, T12	0	18	3	12	21	198	Incomplete
Case 4	127	L1	7	10	5	9	23	163	Incomplete
Case 5	98	T12	9	0	4	10	25	196	Complete
Case 6	3	T12	3	8	3	13.5	24	254	Incomplete
Case 7	111	L1	13	16	5	15	27	318	Incomplete
Case 8	1	L1	21	4	5	10	22	173	Incomplete
Case 9	30	L1	24	7	7	10	24	188	Incomplete
Case 10	2	L3	7	3	3	12	25	236	Incomplete

**Table 3:** One-month follow-up outcomes of patients receiving kyphoplasty for thoracolumbar burst fracture.

	Disposition	Neuro Deficit	Cement Leakage or Extravasation	Spine Surgery	Mobility Improving	Pain Improving	Follow-Up X-Ray
Case 1	home	N	N	N	Y	Y	stable
Case 2	acute rehab	N	N	N	N	N	significant progression of the L2 burst fracture
Case 3	subacute rehab	N	N	N	Y	Y	stable
Case 4	home	N	N	N	Y	Y	stable
Case 5	home	N	N	N	N	N	stable
Case 6	home	N	N	N	Y	Y	stable
Case 7	home	N	N	N	NA	N	stable
Case 8	home	N	N	N	Y	Y	stable
Case 9	home	N	N	N	NA	Y	stable
Case 10	SAR	N	N	N	Y	Y	stable

Abbreviations: NA = not applicable due to not having issues with mobility prior to kyphoplasty/vertebroplasty; N—No; Y—Yes.

## Data Availability

The data presented in this study are available on request from the corresponding author due to Health Insurance Portability and Accountability Act (HIPAA).
